# Is the Life of a Mediocre Philosopher Better Than the Life of an Excellent Cobbler? Aristotle on the Value of Activity in *Nicomachean Ethics* x.4-8

**DOI:** 10.1007/s10790-021-09805-1

**Published:** 2021-04-10

**Authors:** David Machek

**Affiliations:** Institut für Philosophie, Universität Bern, Länggassstrasse 49a, 3012 Bern, Switzerland

## Introduction: Aristotle’s Ethics of Activity

There is a deeply entrenched view among philosophers that Aristotle is the founding father of virtue ethics, this being one of the main currents in contemporary ethics alongside consequentialism and deontology.^1^ But this view has also been criticized or qualified on different fronts both by scholars of Aristotle^2^ and by ethicists.^3^ In particular, it has been pointed out that Aristotle does not hold the view according to which the goodness of virtuous action is fully owed to, and explained by, the virtuous character of the agent; Aristotle might even have been committed to the view that it is possible to do perfectly virtuous actions without being virtuous.^4^ If this interpretation is right, it has an important implication for Aristotle’s understanding of what makes an action or activity intrinsically good: the virtue or excellence of the agent is an important source of an activity’s goodness, but not the only one.^5^

The objective of this article is to propose that the question of whether action can have worth that is independent of the excellence of the agent is best addressed within the broader framework of Aristotle’s views about value of activities in general.^6^ I shall do this by means of a close reading of selected passages from book ten of the *Nicomachean Ethics* (hereafter *EN*), in which Aristotle assigns to “activity” a fairly wide and heterogenous range of value terms: activity can be more or less “perfect,” (*teleia*) “serious,” (*spoudaia*) “esteemed,” (*timia*) or “continuous,” (*suneches*) to mention just some of them. The relevant material spans two different discussions—Aristotle’s theory of pleasure, and his account of the superiority of the contemplative life—but there is a substantial continuity between these discussions, as brought out in particular by the central role of “activity” in them. A striking feature of this stretch of the *EN* is that virtue plays only a marginal role in it; it is just one of a number of attributes that can make an activity intrinsically good. But this does not necessarily signal any discontinuity between book ten and the rest of the *EN*; rather, Aristotle here provides a general account of activity and its value that includes moral actions.^7^ In other words, the possibility of the disconnect between doing morally worthwhile actions and being virtuous is a manifestation of a deeper structural feature of Aristotle’s ethical theory, namely the heterogeneous structure of activity’s value.

It is not virtue, but activity or actualization, that is the most distinctive, innovative and fundamental feature of Aristotle’s ethical theory in the context of ancient Greek thought. Aristotle emphasizes that what ultimately matters is not the mere possession of excellence but its active exercise.^8^ This is why Aristotle says that “activities control life” (*hai energeiai kuriai eisin tês zôês*).^9^ This means that activities are the basic building blocks of our life, and that they are the ultimate bearer of value: other good things besides activities are good only insofar as they make activities good, or, as Aristotle puts it, make them “productive of happiness” (*eudaimonikos*).^10^ This holds especially for virtue, since happiness is defined as “activity in accordance with virtue” (*kath’aretên energeia*). This implies that virtue is necessary for happiness, but not that it is sufficient for it, nor indeed that activities that fall short of excellence cannot have a significant virtue-independent worth.

Insofar as activity is the final value-bearer, we would expect that moral choice (*prohairesis*) can be parsed as a choice of activities, or a choice among activities. And indeed Aristotle does sometimes explicitly describe choice in these terms. On the largest possible scale, the choice that can—at least theoretically^11^—be made is the choice among different kinds of lives: that of politics, that of philosophy, and that of enjoyment.^12^ Since life is an activity,^13^ this amounts to a choice between different activities. Another example of choice between activities would be a choice between doing a single “fine” (*kalos*) action at the cost of sacrificing one’s life on the one hand and the activity of living “indifferently” (*tuchontôs*) for many years on the other.^14^ In both these cases, Aristotle presupposes that activities can be objectively compared and ranked according to their value: the political life is better than the life of pleasure, but it is trumped by the philosophical life; and the value of a short-lived but fine activity exceeds the value of a longer-lived but mediocre activity.

But how precisely are we supposed to parse the value of activity and justify these choices? What are the different considerations that go into the determination of activity’s value, and how are we to weigh them against one another? And what role does the virtue or excellence (*aretê*) of activity play in this determination? *Prima facie*, at least, it seems that excellence cannot play any role in justifying the superiority of the life of contemplation over the political life; the contemplative life cannot be said to be, by itself, more excellent than the political life. In the second case, we can find the role for excellence more easily: the “fine” action will, presumably, be also excellent. But it does not necessarily follow either that activity’s value is exhausted by being done out of excellence, or indeed that excellence is the necessary condition for doing fine actions—can it not happen that even a non-virtuous person should finely sacrifice her life? I shall try to determine, then, how far the material in *EN* X.4-8 can help us in answering these questions.

This interpretation will be of value both for scholars of Aristotle and for ethicists who are interested in the legacy of Aristotle’s ethics in the contemporary context. As of yet, we do not have a systematic discussion of Aristotle’s axiology of activity. Given that activity plays such a central role in Aristotle’s ethics, an attempt to categorize and compare different components of activity’s value is bound to enhance our understanding of Aristotle’s account of the good life, and, in particular, of the value of different kinds of human life. From the contemporary perspective, the contribution of this interpretation is that it appreciates the centrality of those features of Aristotle’s theory that make him a proponent of perfectionism, rather than virtue-ethics. On the perfectionist view, goodness of actions or activities derives not, or not only, from the virtuous character of the agent, but rather from the kind or type of pursuit that this activity belongs to. Aristotle has been sometimes interpreted in the contemporary scholarship as a perfectionist, particular in the works of Thomas Hurka. The present interpretation can give further support to this view from the perspective of Aristotle’s axiology of activity.

The structure of the paper is as follows. I start in Part Two by proposing how to categorize the different value-conferring attributes of activity in *EN* X.4-8. I suggest that we can distinguish between activity’s excellence and its excellence-independent worth. The latter can then be divided into two broad kinds, the value related to an activity’s worthwhileness and the value related to an activity’s continuity. In Part Three, I move beyond the strict textual interpretation and discuss what this categorization implies for the causal relationship between these kinds of value. Aristotle’s discussion of contemplative activities in *EN* X tends to suggest that these attributes typically go hand in hand, but on a closer inspection their interdependence appears to be questionable. Whereas these kinds of value often promote each other, they can arguably also occur in isolation, and in some cases can even compete with each other or occur at each other’s expense. In Part Four, I shall argue that the above analysis of activity’s value is, with some modifications, also applicable in the practical realm of moral actions, and that it has specific consequences for our understanding of what makes these actions valuable. Finally, in Part Five I turn to the question of how to weigh different components of activity’s value, and how to rank different activities—and lives—depending on their participation in these components. Whereas Aristotle does not give an explicit answer to these questions in his extant texts, I shall make some suggestions on Aristotle’s behalf based on available textual clues.

## Worthwhileness and Continuity as Excellence-Independent Aspects of Activity’s Value (*EN* X.4-8)

When reading Aristotle’s discussion in *EN* X.4-8, the first aspect of activity’s goodness or perfection is what we can call its *excellence* in the sense of how well or poorly the activity is executed. In this sense, Aristotle talks about excellent seeing, excellent exercise of geometry, or excellent cithara-playing. Excellence of activity can depend on the external circumstances, such as light or darkness in case of seeing, but it is conferred primarily by the good condition of the corresponding capacities (*dunameis*) or dispositions (*hexeis*) of the soul. So, for instance, the activity of seeing will be “best” (*beltistê*) or “most perfect” (*teleiotatê*) when those who see are in the “good condition” (*eu dikaiomenos*), i.e., when they are able to see well.^15^ In the case of doing geometry, the activity will be excellent in the presence of geometrical expertise. Pleasure in an activity is typically conducive to its excellent performance insofar as it optimizes the exercise of relevant capacities, so that the experts in geometry will be more “precise” (*akriboi*) because they enjoy their activities.^16^ Later in *EN* X, Aristotle says that the source of “excellent activities” (*spoudaiai energeiai*) is “excellence (*aretê*) and understanding (*nous*).”^17^ This agrees with the view expressed in *EN* I that the performance of cithara-playing becomes “excellent” (*spoudaia*) when its practitioner has “excellence” (*aretê*) in the sense of good cithara-playing skills.^18^

But activities can also derive their value from a different source than excellence, namely from their excellence-independent worth. Whereas excellence refers to how an activity is done, worth derives rather from what we do, from what kind of pursuit this activity is. We could also say that the source of excellence is in the agent, whereas the source of worth is rather in the activity itself. This is, presumably, what Aristotle has in mind when he says that some activities, in particularly the activity of contemplation, are “intrinsically estimable” (*kath’autên timia*).^19^ Perhaps the most salient and frequently used term that refers to the intrinsic worth of activity is *spoudê*, “seriousness,” “worthwhileness” or “worthwhile seriousness.”^20^ As some of the above quotations bring out, the word *spoudaios* sometimes has the meaning “excellent” in the above sense of impeccable performance. But in some contexts this word clearly means “serious” or “worthwhile” and not necessarily “excellent.” Consider the following claim that intellectual activities are better because they surpass political activities in *spoudê*:

If, then, among actions in accord with the virtues, those in politics and war stand out in nobility and magnitude but these are unleisured and seek some end rather than being choiceworthy because of themselves (*di’autas airetai*), whereas the activity of understanding seems to be superior in seriousness (*spoudêi diapherein dokei*) because it is contemplative, to seek no end beyond itself, and to have its own proper pleasure ... .^21^

Reeve, whose translation I otherwise follow, translates *spoudêi diapherein* as “superior in excellence”, rather than “superior in seriousness” or “superior in worthwhileness.” But this translation is questionable, insofar as the quality that distinguishes the intellectual activities is not the excellence of performance—for it seems clearly possible that intellectual activities could be exercised poorly, where one lacks intellectual excellence—but rather a worth that derives from attributes of activity other than excellence. This worthwhileness has three different sources. One is the autotelic character of worthwhile activities, i.e. that they “seek no end beyond themselves” or “are choiceworthy because of themselves.” This autotelic character usually implies that we take pleasure in these activities, which is typically the case with activities of “leisure” (*scholê*).

Another source of activity’s worthwhileness is the worth of the part of the soul whose actualization the activity is. Aristotle says that “in every case the activity of what is better, whether a part or a human being (*tou beltionos aei moriou kai anthrôpou*) is more worthwhile (*spoudaiotera*).”^22^ An activity can become more worthwhile either due to the excellence of the agent or because it is an actualization of a worthier part of the soul. This psychological ranking is fourfold, from the least to the most worthy: the nutritive part of the non-rational part of the soul; the reason-responsive part of the non-rational part of the soul; the practical part of the rational part of the soul; and the theoretical part of the rational part of the soul.^23^ The worthwhileness that derives from the worth of the respective part of the soul does not depend on excellence: the fact that an activity actualizes the rational rather than the non-rational part of the soul seems to have no bearing on whether the activity is excellent or not. So even if such a rational activity lacks excellence, it is presumably still not worthless.

Finally, a cognitive activity can presumably derive a portion of its worthwhileness from the seriousness of its objects: “the most perfect is the activity of a subject that is in good condition in relation to the most serious (*pros to spoudaiotaton*) of the relevant objects.”^24^ We may plausibly speculate that Aristotle’s remark about activity’s “purity” (*kathareiotês*) can be understood along these lines: “Sight differs from touch in purity, however, as hearing and smell do from taste.”^25^ Aristotle does not make clear what the purity of activity depends on, but it may well be the purity of its object; “purity” certainly reminds us of Plato and his forms as the purest objects of cognition. Thus, sight would be purer than hearing on the grounds of the greater purity of its objects, which makes a good sense insofar as the sight provides access to the ideal objects of geometry.

Thus, altogether there seem to be three main worthwhileness-conferring attributes: having the end in itself; worth of the psychological capacity; and the worth (or purity) of the object. But we should note that according to these criteria, even some intellectually demanding pastimes would qualify as rather worthwhile activities: playing chess, for instance, is an intellectual, autotelic activity, insofar as we play chess for the sake of it. And yet Aristotle would not regard such an activity as having a significant degree of worthwhileness. For the serious (*spoudaia*) activities are opposed to the “ridiculous” (*gellaia*) activities that we do for the sake of amusement or relaxation (*anapausis*). Insofar as we relax in order to be able to accomplish serious things, and not the other way round,^26^ the activities of amusement are never autotelic in the strict sense.

Excellence and worthwhileness do not seem to exhaust the entire scope of activity’s value. For in the course of *EN* X Aristotle also introduces a cluster of activities’ attributes such as “self-sufficiency,” (*autarkeia*) “continuity,” (*sunecheia*) or being “unweary” (*autruton*) and “long-lasting” (*chronios*). Looking outside of *EN* X, the attribute of “being free from impediments,” (*anempodistos*)^27^ or the quality of being “steadfast” or “lasting” (*monimos*)^28^ would also belong here. Continuity is not quite the same thing as long-lastingness; and neither of these is quite identical with steadfastness or freedom from impediments. For the sake of convenience, and for the lack of a better word, I shall collectively refer to these attributes simply as “continuity-related” attributes or more crudely as “continuity.” Continuity-related notions constitute a component of an activity’s value that belongs to the category to excellence-independent worth and yet is different from worthwhileness. In contrast to worthwhileness, continuity-related attributes do not, by themselves, command esteem or respect, or do not deserve praise or blame. And yet they are also clearly different from, and to some extent independent of excellence.

Some of these attributes are related to the discussion of pleasure: for instance, pleasure is defined as “unimpeded activity,”^29^ and pleasure tends to make activities more long-lasting;^30^ some are related primarily to the account of contemplative activities, for these are said to be particularly continuous and self-sufficient.^31^ But all these attributes are rather closely intertwined: for instance, an activity tends to be more long-lasting when it is more self-sufficient, i.e. less dependent on external resources and circumstances. All these attributes make the activity that has them more god-like,^32^ insofar as impediments, fatigue and dependence on material resources belong to the human predicament. Given that the gods are “blessed and happy to the highest degree,”^33^ these attributes make the activity more choiceworthy.

So far, I have been arguing that the different value terms that Aristotle attributes to activities can be grouped into three logically different categories. But this has by itself no implications for the causal relationship between these categories, i.e. for the question whether they are coextensive, whether they mutually entail each other like the virtues do (at least according to Aristotle), or whether they are interdependent only in some weaker sense. As far as the contemplative activities are concerned, there is an obvious intention on Aristotle’s side to suggest that these activities are intrinsically such that these different kinds of value converge in them. They tend to be most worthwhile, most continuous and also most excellent. The implication is that there is nothing to lose in dedicating one’s lifetime to these activities, in comparison to other pursuits. But it is far from clear whether this is the accurate account of the relationship among these different categories. In the following section, I shall offer a more skeptical assessment.

## Causal Relationships Between Activity’s Excellence, Worthwhileness, and Continuity

We can start by appreciating the extent to which there is a synergy among excellence, worthwhileness and continuity. This synergy is owed to the fact that different categories of value have in part identical sources. The most obvious case is the relationship between excellence and continuity: pleasure in an activity enhances both the activity’s excellence and its continuity. The better one is at an activity, the more one tends to enjoy it, which, in turn, makes the activity increasingly immune to fatigue and distractions;^34^ conversely, the longer and the more focused one’s activity is, the better or sharper one’s performance tends to become, both within a single episode of performance and over time.^35^

There is a similar synergy between continuity and worthwhileness, but it is more one-sided. Worthwhileness promotes continuity, insofar as the attributes that make an activity worthwhile also tend to make it more continuous or long-lasting. This follows from Aristotle’s view that the activities of the mind are on the whole much less fragmented than the activities of the less worthy capacities of the soul, or that the more serious objects tend to make activities more truly pleasant, and thus more continuous. But the effect does not seem to work the other way round. The attributes that make activity continuous—i.e. persistent motivational focus, immunity to distractions, resistance to fatigue etc. —won’t, by themselves, increase its seriousness.

It is somewhat more difficult to find any such synergy in the relationship between excellence and worthwhileness. The ambiguity of the Greek word *spoudê* or *spoudaios*, which remains unresolved in most English translations, induces the impression that the contemplative activities are at once most worthwhile and most excellent. Consider the translations of Aristotle’s above-cited claim “that in every case the activity of what is better, whether a part or a human being (*tou beltionos aei moriou kai anthrôpou*) is more excellent (*spoudaiotera*).” Rowe has “more serious;”^36^ Irwin “more serious and excellent,”^37^ similarly to Crisp’s “more serious, more virtuous.”^38^ But to be quite precise, we should say that the activity is more serious, or worthwhile, insofar as it is the activity of the worthier part of the soul; and that it is more excellent to the extent that it is the activity of a better, or more excellent, person. These two meanings of *spoudaios* appear to have little in common in substance. 

Indeed, one can go quite far in dissociating excellence from worthwhileness. Consider, for instance, Aristotle’s discussion of the pastime activities of amusement. One can no doubt achieve a considerable level of excellence in some of these activities, e.g. trivial games, but that won’t necessarily compensate for their lack of worthwhileness. We can also take another type of activity which Aristotle explicitly connects with the category of “excellence,” (*aretê*) namely technical activity.^39^ Even though these activities presumably have a degree of worthwhileness, insofar as they engage the rational part of the soul, they are on the whole inferior in worthwhileness to contemplative (or indeed political) activities. But they can be superior in excellence to activities that exceed them in worthwhileness: the activity of a virtuoso cobbler will fall short in worthwhileness compared to the activity of a mediocre metaphysician, but it will beat it in excellence.

A considerable disconnect also looms between continuity-related notions on the one hand and both excellence and worthwhileness on the other. An activity can have a steady flow even if it is fairly deficient in excellence and worthwhileness. For instance, one can arguably indulge in a worthless as well as poorly performed activity with considerable focus and persistence, and over a significant period of time; think of a mediocre player of primitive video games. Vice versa, we can imagine cases of activities that are both excellent and worthwhile, such as an excellent pursuit of metaphysics, and yet are deficient in continuity, whether on account of agent’s poor health, or due to adverse external (e.g. political) circumstances.

The picture of the relationship between different kinds of activity’s value could depart even further from the ideal of ultimate convergence if there is a competition among these kinds. Could it be the case that we sometimes have to choose an activity’s excellence at the expense of its continuity, or trade off its excellence for a portion of its worthwhileness? It seems that Aristotle himself is aware of this possibility when he says that the virtuous person will be willing to sacrifice “many years” of her life for the sake of accomplishing a grand noble deed (see above). In light of the above discussion, we could parse this as a trade-off between a lasting activity inferior in worthwhileness, for a short-lived activity superior in worth. In this case, the dilemma may be solvable without any significant cost for Aristotle’s theory. It requires only an admission that there are situations in life when you cannot have it all, and that even a virtuous action, insofar it comes at the expense of a premature end of life, may paradoxically be the one that indirectly deprives one of happiness.^40^

But it is possible that there are other cases of trade-off that are potentially more challenging from the perspective of Aristotle’s theory. There are reasons to doubt that the contemplative life, despite being filled with the most worthwhile activities, will for that reason be automatically positioned to score highly in terms of excellence and continuity. Indeed, the high level of its worthwhileness, far from promoting excellence and continuity, can make it more difficult for the contemplative life to achieve them. The activity of contemplation, or abstract thought, makes especially high demands on one’s psychological resources, particularly on one’s capacity to concentrate, and is thus arguably more vulnerable to fatigue and disruption than many other cognitively less demanding activities. If one should aim primarily at an activity’s duration and continuity, one would be better advised to choose other kinds of activities, say low-impact gardening. The same can be said about excellence. It is arguably more difficult to achieve excellence in contemplative activities than in many other less worthwhile activities. Unless one is both in the socially privileged position to afford the necessary education (as the audience of Aristotle’s lecture presumably was) and has the rare talent needed to achieve excellence in this kind of pursuit (as Aristotle’s audience might not necessarily have been), one would do better to choose a different kind of pursuit if excellence is the main goal.

## Value of Moral Actions

Aristotle’s discussion of activity and its value in *EN* X is concerned with activities that are not morally salient: activities like seeing, listening to the flute or doing geometry cannot be assessed on account of their virtuousness or viciousness. Indeed, when Aristotle does talk about one’s activity being virtuous or vicious, he typically talks about “action” (*praxis*) rather than “activity” (*energeia*). He once uses the phrase “actions and activities,” (*praxeis kai energeiai*)^41^ which suggests that there is a difference between the two, and even raises the question whether actions are to be discussed on the same level as activities. As Sarah Broadie has noted, it is not activities but actions that “really express our ethical nature.”^42^ And when we think about moral value, then action is a far more suitable vehicle for that value than activity. Activities are, ultimately, actualizations of our psychological capacities, something that happens in our soul. But actions happen, or rather are done or accomplished, in the world. When we praise somebody for having accomplished a noble deed, we do not really praise them for whatever psychological process took place in their soul, but for what they have done. Moreover, there is an important difference in the temporal structure of activities and actions. It is characteristic for activities that they have a certain temporal extension, whereas actions seem to be accomplished at a particular point in time: “there seems to be no such thing as the *activity* of paying a debt.”^43^ So there is a worry that whatever implications can be drawn from Aristotle’s discussion of activity’s value, they do not really apply to the realm of actions.

But there are clear indications that Aristotle does not mean to exclude moral actions from his discussion of activity in *EN* X. When Aristotle talks about political “activities” as “actions,” he directly compares their value with the contemplative “activities.”^44^ In the explicitly moral contexts, he uses “activity” in a sense that is clearly interchangeable with “actions.”^45^ Indeed, it would be odd if actions turned out to be substantially different from activities if the human end is defined as “activity in accordance with virtue.” So there are strong reasons to think that actions are special kinds of activity or that every action has its corresponding activity or activities. Even the action of paying a debt can be traced to the underlying actualization of certain psychological capacities responsible for making the corresponding choice and carrying it out.

What distinguishes actions from other kinds of activities is a peculiar relationship to their *ergon*, i.e. the deed. At the very beginning of the *EN*^46^ Aristotle distinguishes between two kinds of activities: those that do not have an end beyond themselves, and those that do. In *EN* VI.5, he says that acting well (*eupraxia*) is itself the end, in contrast to technical activities that aim at a product beyond themselves. But then again, in *EN* X.7 he expressly says that actions do seek an end beyond themselves, in contrast to the contemplative activities. These claims can be reconciled if we take into account the difference between practical activity and the deed it accomplishes. When Aristotle says that good action is itself the end he does not have in mind the corresponding activity that underlies the action, but the virtuous deed that is accomplished. This is quite consistent with the view that, in contrast to contemplative activities, practical activities indeed have their end beyond themselves, namely in their deed: it is our aim to actually pay the debt, help the friend—i.e. to produce a change in the world—and not merely to actualize certain capacities of our soul in a certain way.

This account of the relationship between action’s activity and action’s deed raises the question: what makes an action good? Is it the psychological activity, is it the deed accomplished, or both? I argued in the introduction that the activity is the ultimate value bearer in Aristotle’s theory. So when it comes to action, it is in the underlying psychological activity where the value of the entire action should be located. This does not mean, of course, that the value of the resulting deed is indifferent; it is rather that the deed, if it is good and if it is accomplished, confers an extra value on the activity from which it results, in comparison to an activity that fails to accomplish the deed it aims at.

*Prima facie*, however, there are grounds to think that the classification of different aspects of activity’s value reconstructed from *EN* X does not in some important respects fit the special but important kind of morally salient activities that underlie moral actions. Firstly, at least some of the “continuity-related” attributes do not seem to play any significant role in the moral context. It is unclear why activity’s continuity or long-lastingness should be of any value. Rather, these activities should last precisely only as long as is necessary to accomplish the moral deeds they aim at. Secondly, and perhaps more importantly, the separation of excellence and excellence-independent worth is deeply problematic in the case of moral actions. For one thing, the notion of moral excellence is by definition quite different from the morally unspecific notion of excellence in the sense of perfect execution. The latter is morally indifferent; even a thief can be excellent at what he does.^47^ This moral indifference makes it possible to neatly separate the purely technical or aesthetic value of *how* we do something from the value of *what* we do. But in the moral realm such a clear separation is clearly problematic; it is built into the very notion of moral excellence that one can only act virtuously insofar as one does virtuous actions. The goodness of how one acts presupposes the goodness of what one does. Vice versa, Aristotle makes remarks that suggest that the actions have moral worth only if they are performed with a moral excellence that inheres in the character of the agent: “for actions in accord with the virtues to be done temperately or justly it does not suffice that they themselves have the right qualities; rather the agent must also be in the right state when he does them.”^48^ If one cannot do virtuous action without being virtuous, then the notion of excellence-independent worth in the moral realm is undermined.

On a closer look, though, the general axiology of activity can be extended even to the special case of moral actions, provided we are ready to adjust the axiological scheme so that it can accommodate some distinctive features of these actions. As far as continuity-related attributes are concerned, we can say that they do have a value but this value is conditional upon the success of the activity: an activity that is free from disruptions and impediments is better than an activity of precisely the same kind, insofar as the absence of impediments or disruption is conducive to the accomplishment of the relevant virtuous deed.

When it comes to the relationship between moral excellence and moral worth, we should say that the undeniable fact that there is no moral excellence that can be achieved independently of moral worth does not entail that there cannot be moral worth that can be achieved independently of moral excellence. In fact, the existence of excellence-independent moral worth is possible if “an action is virtuous in virtue of objective features it has independent of the motivations of the agent performing the action.”^49^ Aristotle hints most suggestively at this possibility when he draws an implicit distinction between doing virtuous actions, i.e. performing activities with moral worth, and acting virtuously, i.e. performing activities with moral excellence.^50^ The precise account of what defines the excellence-independent moral worth of actions has been a matter of scholarly debate. For the sake of the present discussion, I take onboard the account according to which an action is virtuous if it “aims at good ends.”^51^

The possibility of excellence-independent moral worth is implied, or presupposed, in several aspects of Aristotle’s moral theory. One is his account of “habituation” (*ethismos*). As has been argued, in particular by Marta Jimenez,^52^ this theory presupposes that it is possible to do virtuous actions prior to becoming virtuous. One can do virtuous actions prior to becoming virtuous by chance or because one follows the instructions of one’s educators. On this view, habituation can be described as a process in which the habituees become better by doing actions that are better than themselves. We can note that this may also be an accurate description of projects or pursuits that do not necessarily have to do with moral development. Think of the activity of parenting. According to Aristotle, parents have “conferred greatest benefits on children,” including, first and foremost, the fact of bringing them to the world.^53^ Assuming that the action that involves conferring a benefit on another person has moral worth, then the very act of becoming and being a parent will be morally worthwhile even in cases when the parents themselves are not virtuous. Indeed, there are many non-virtuous parents, as well as parents that fall short of being excellent parents, whose parenting still commends considerable respect insofar as by nurturing their children and bringing them up they accomplish something at least modestly fine. The moral worth of their activity does not wholly depend on their moral excellence, but is in part also conferred by the nature of the pursuit in which they have become involved.

More evidence in favor of the possibility of excellence-independent moral worth is suggested by Aristotle’s account of self-controlled persons (*enkrates*). For the self-controlled can not only make right decisions, i.e. choose virtuous actions for their own sake and for the right reasons, but also realize them, i.e. bring about the deed decided upon.^54^ Even though their motivational state will fall short of that of virtuous persons—for their desire for the good, and their pleasure in acting finely, will be tainted by their pain in having to forego the vicious pleasure—they will typically accomplish the same virtuous deeds as virtuous persons.

The separation of moral worth from moral excellence can be traced even deeper into the heart of Aristotle’s ethical theory, namely to his account of the human function (*ergon*) in *EN* I.7. In order to arrive at the definition of the chief good of humans, we have to identify their characteristic function or activity: for the chief good will reside in this function. Aristotle defines the function of a human being as “activity of soul in accordance with reason, or not apart from reason”^55^ or as “activity of soul and actions accompanied by reason.”^56^ From the perspective of the axiology of activity from *EN* X, the characteristic activity of humans is to be identified according to the criterion that confers worthwhileness; for the characteristic activity is defined with regard to the part of the soul whose actualization it is. Note that excellence does not play any role in identifying this characteristic activity. Aristotle does not say that the human function amounts to an excellent exercise of reason-related activities, but rather that the human good, i.e. the excellent exercise, *resides in* this function (*en tôi ergôi*).^57^ As in the case of a cithara-player, the human function can be exercised well or badly. If it is exercised well, we achieve happiness; if it is exercised badly, we remain unhappy, but that does not necessarily detract from the fact that we still exercise our human function.^58^ Even vicious humans still exercise the characteristic human activity, for vicious action still entails a high level of distinctly human rationality, insofar as it springs from “rational choice” (*prohairesis*).^59^

Thus, the procedure by which Aristotle arrives at his account of happiness consists of two clearly differentiated steps. Firstly, he defines the broad type of relevant activity, or says *what* we have to do if we want to live qua humans. Secondly, he adds another criterion, namely *how* we need to do these kinds of activities, i.e. excellently, if we want to achieve the human good. The first step refers to the category of worth, the second to the category of excellence. Aristotle himself indicates that there is a difference between these two levels when he says that an excellent cithara-player is defined when excellence is “added” (*prostithemenês*) to the function:^60^ firstly, it has to be someone who does the relevant kind of activity, i.e. plays cithara; secondly, it has to be someone who does this kind of activity with excellence.

One important problem raised by this interpretation of the function argument is the worth of vicious activities. For, by Aristotle’s own standards, these activities could in fact have a considerable worth. Think of a vicious politician. Insofar as his actions spring from activities that involve the rational part of the soul, they have some degree of worthwhileness to start with. If, in addition, he chooses these activities for their own sake, which is precisely what the vicious characters do, the seriousness of these activities is further enhanced. Finally, this worthwhileness is not even tainted by childishness or ridiculousness; for this politician is serious and consistent in what he does. And yet we feel that Aristotle would hardly deem any vicious life commendable, not even relative to the lives of those who are permanently in a vegetative state, or the lives of seriously deformed humans, such as human brutes, who do not have rational parts of the soul at all.^61^ Is this difficulty yet another indication that morally salient activities do not fit the axiological categorization extracted from *EN* X? Not necessarily, for it is possible to maintain that the vicious actions do have some degree of worthwhileness, and yet that they are, on the whole, not worth choosing. It suffices to say that the moral dis-worth that they have on account of aiming at bad ends outweighs any worth that they have on account of involving the exercise of rationality.

## Ranking Lives by Weighing Different Components of Activity’s Value

The overall value of activity depends on its degree of excellence, worthwhileness and continuity. But to compare different activities in terms of their overall value, we need to know how to weigh these different components. Think of an activity A that has a 9/10 excellence, 9/10 continuity, but only 1/10 worthwhileness; and an activity B that has a 1/10 excellence, 5/10 continuity, and 9/10 worthwhileness. Which of these two activities will be more worth choosing, *all things considered*? It is fair to say that it is impossible to glean a comprehensive algorithm for weighing different components of activity’s value from Aristotle’s extant texts. But it is perhaps possible to use the existing clues to sketch, in broad terms, how Aristotle might answer this question. I shall do so by returning to the theme of choosing different lives mentioned in the introduction.

In *EN* I.5, Aristotle distinguishes three “prominent” kinds of lives: the life of pleasure, the political life and the contemplative life. In *EE* I.4, he mentions, in addition, lives “devoted to vulgar trades, and to commerce, and the banal occupations.”^62^ We know how Aristotle ranks these lives: the contemplative comes first, closely followed by the political life; the life of pleasure clearly comes last. As for the life of banausic occupations, where the life of a cobbler or a peasant would belong, this too does not “even enter the contest for good fortune.”^63^ Whereas this is never made quite explicit, the primary criterion of this ranking is the worthwhileness of these activities. The contemplative life is best because it actualizes the best part of the soul, the political life comes second because it actualizes the second-best part of the soul, and the life of pleasure is last among prominent lives because it is an overwhelmingly non-rational life. The life devoted to banausic pursuits is even less worthwhile, because it is aimed at providing for the necessities of life. This means, presumably, that in comparison to other lives it is devoid of activities that have their end in themselves. Given that these banausic lives may consist of overwhelmingly rational activities (as is particularly the case in the lives of craftsmen), this ranking implies that, in terms of worthwhileness, the autotelic character of activities matters more than their psychological worth.

We can now ask whether this basic ranking would change if we factor in the excellence of these lives. The philosophical life is better than the political life; but what about the life of an excellent politician in comparison with the life of a mediocre philosopher? To make matters even more complicated, we can also add the continuity-related attributes. One life may be better than another because it is unruffled, free from difficulties and impediments, less fragmented, or simply because it lasts longer. Supposing that the life of an excellent politician is, on the whole, better than the life of a mediocre philosopher; would the former still trump the latter if it happens to be long and free from impediments, whereas the latter happens to be fraught with hardships?

What seems relatively clear is that among the three components, the worthwhileness is the most fundamental. For no matter how much excellence or freedom from impediments a life may have, that excellence or freedom will matter only on the condition that this life consists of worthwhile activities in the first place. This seems to follow from the account of the human function. A banausic life that is unruffled and long is not even a bit closer to happiness than a banausic life that is short and difficult, because it does not consist of the kind of activities that are constitutive of the human happiness; its value does not ever get off the ground. But once a life is worthwhile in some minimal sense, then the addition of excellence could actually make a significant difference. Consider the life of an excellent politician that scores, say, 8/10 in worthwhileness and 10/10 in excellence, and the life of a mediocre philosopher that scores 10/10 in worthwhileness but only 5/10 in excellence. By Aristotle’s standards, the life of this politician would surpass the life of this philosopher, because the former would count as a happy life insofar as it consists in the excellent exercise of function-fulfilling activity, whereas the latter would ultimately fall short of happiness due to falling short in excellence.

Suppose now, further, that the life of the mediocre philosopher happens to be long, and free from major impediments and disturbances, whereas the life of the excellent politician is fraught with setbacks and hardships, and perhaps even ends in a noble but premature death. Would the political life still remain superior? According to Aristotle, it probably would. On the one hand, severe impediments of activities do diminish the blessedness of virtuous life (1100b29-30).^64^ On the other hand, Aristotle argues that the good person would rather choose a single grand noble deed over many lives lived indifferently (see above), which suggests that, if a trade-off is necessary, the continuity-related attributes will not ever beat excellence and worthwhileness. Severe impediments may deprive the virtuous person of blessedness, but this life will still have a considerable overall value, insofar as it is a noble life (1100b30-32).^65^ We might doubt that the same could be said about the life of a mediocre philosopher, no matter how continuous it may be. This conclusion does not imply, though, that the continuity-related attributes cannot make a significant difference in the activity’s overall value. With considerations of worthwhileness and excellence being judged equal, a life which is more continuous will presumably beat the life which is not.

## Conclusion

In the *Nicomachean Ethics* Aristotle introduces a broad and fairly diverse catalogue of terms in connection with the value of activity. I have drawn attention to the fact that, on his view, activities can owe their goodness not only to the excellence of the agent, but also to some of their intrinsic qualities that are wholly or partly independent of the agent’s virtue or goodness. In particular, an activity’s worthwhileness is an important happiness-conducive aspect of its value that does not depend on the activity’s excellence, and can in principle even compromise it. The structure of activity’s value, as implied by Aristotle’s account, is fairly heterogenous, which implies that different components of activity’s value must be sometimes weighed against each other if we are to rank different activities according to their overall value. Such a ranking is necessary in situations when we have to make an informed choice between two or more activities. Perhaps the most important of these choices among activities, and one that Aristotle himself discusses, is the choice of one’s entire life. On the whole, Aristotle would no doubt advise us to choose lives that can score best on all three counts. If this is impossible and hard choices are necessary, he would urge us to prioritize the considerations of worthwhileness, or *what* we do. It is the kind of pursuit that is the most decisive. The value conferred by *how* we do it, or excellence, comes next, so that in some cases, perhaps, a great increase in excellence could reasonably be traded for a small decrease in worthwhileness. The value conferred by continuity-related attributes comes last: whereas they do add further value, this increase will not generally affect the ranking based on excellence and worthwhileness.

## Footnotes

1 See, for instance, Rosalind Hursthouse, *On Virtue Ethics* (Oxford: Oxford University Press, 2000); Julia Driver, *Ethics: The Fundamentals* (Oxford: Wiley-Blackwell, 2006).

2 See Richard Kraut, *Aristotle on the Human Good* (Princeton: Princeton University Press, 1989); Joachim Aufderheide, “Is Aristotle a Virtue Ethicist?” in V. Harte and R. Woolf (eds.), *Rereading* *Ancient Philosophy* (Cambridge: Cambridge University Press, 2017), 199–220; Sukaina Hirji, “What’s Aristotelian about neo‐Aristotelian Virtue Ethics?”* Philosophy and Phenomenological Research* 98.3 (2019): 671–696.

3 See Thomas Hurka, *Virtue, Vice and Value* (Oxford: Oxford University Press, 2000); Christine Korsgaard, “From Duty and For the Sake of the Noble: Kant and Aristotle on Morally Good Action” in A. Cohen (ed.), *Kant on Emotion and Value* (Basingstoke: Palgrave Macmillan, 2014), 33–68.

4 Marta Jimenez, “Aristotle on Becoming Virtuous by Doing Virtuous Actions.” *Phronesis* 61.1 (2016): 3–32; Terence Irwin, “Aristotle’s Conception of Morality.” *Proceedings of the Boston Area Colloquium* 1 (1985): 115–127; Sukaina Hirji, “What’s Aristotelian about neo‐Aristotelian Virtue Ethics,” op.cit.

5 I mean here goodness in the broadest possible sense; activity is good insofar as it is conducive, in some sense, to happiness.

6 “Action” is a kind of “activity,” or is closely associated with it, as I shall argue in the fourth part of this article.

7 The relationship of this book to the rest of the work has notoriously been found difficult and puzzling; in particular, it has been a matter of debate how, and whether, the firm and explicit commitment to the supreme value of the theoretical or contemplative life can be squared with the extensive focus on, and valorization of, the practical or political life in other parts of the work. See for instance Richard Kraut, *Aristotle on the Human Good* (Princeton: Princeton University Press, 1989); Gabriel Richardson Lear, *Happy Lives and the Highest Good. An Essay on Aristotle’s Nicomachean Ethics* (Princeton: Princeton University Press, 2004). I shall not enter into these debates, and merely note that just as the account of pleasure in this book is supposed to apply generally to all kinds of pleasures, not only those in contemplative activities, so does the account of activity apply to all kinds of activities, including moral actions.

8 See Aristotle, *Nicomachean Ethics*, transl. C.D.C Reeve (Indianapolis: Hackett, 2014), 1098b32–1099a7 and 1070a18-19.

9 Aristotle, *EN*, op. cit., 1100b33-34. Aristotle makes this remark where he argues that no blessed person can ever become utterly wretched on account of external misfortunes; what matters is not what he has, or what he suffers, but what he does, in the broadest possible sense of the actualization of psychic capacities.

10 Aristotle, *EN*, 1099b3, 1176b16 and 1177b23.

11 “[Lives] chosen by all who have the means to do so.” Aristotle, *Eudemian Ethics*, trans. Michael Woods (Oxford: Clarendon Press, 1982), 1215a35.

12 Aristotle, *EE*, op. cit., 1215a20–16b25; Aristotle, *EN*, 1095b14-96a10.

13 Aristotle, *EN*, 1175a12.

14 Aristotle, Ibid., 1169a22-27. Strictly speaking, Aristotle does not talk here about “activities” but “actions” (*praxeis*). But actions are kinds of activities, as I shall argue in section 4.

15 Aristotle, *EN*, 1174b15 ff.

16 Aristotle, Ibid., 1175a31-35.

17 Aristotle, Ibid., 1176b16-19.

18 Aristotle, Ibid., 1098a10-13

19 Aristotle, Ibid., 1178b31.

20 See Joachim Aufderheide, *Aristotle’s Nicomachean Ethics Book X: Translation and Commentary* (Cambridge: Cambridge University Press, 2019), p. 160.

21 Aristotle, *EN*, 1177b17-21.

22 Aristotle, Ibid., 1177a3-6.

23 Here I follow Aufderheide, *Aristotle’s Nicomachean Ethics Book X: Translation and Commentary* (Cambridge: Cambridge University Press, 2019), p. 161, where the references to the relevant bits of *EN* can be found.

24 Aristotle, *EN*, 1174b21-23.

25 Aristotle, Ibid., 1175b36–1176a1.

26 See Aristotle, Ibid., 1176b35–77a1.

27 Aristotle, Ibid., 1153a15.

28 Aristotle, Ibid., 1100b14.

29 Aristotle, Ibid., 1153a15.

30 Aristotle, Ibid., 1175b15.

31 See Aristotle, Ibid., X.7-8.

32 Aristotle, Ibid., 1178b28.

33 Aristotle, Ibid., 1178b10.

34 Aristotle, Ibid., 1175b5 ff.

35 Aristotle, Ibid., 1175a31-36.

36 Sarah Broadie and Christopher Rowe, *Aristotle: Nicomachean Ethics: Translation, Introduction and Commentary* (Oxford: Oxford University Press, 2002), p. 250.

37 Terence Irwin, *Aristotle: Nicomachean Ethics* (Indianapolis: Hackett, 1999), p. 163.

38 Roger Crisp, *Aristotle: Nicomachean Ethics* (Cambridge: Cambridge University Press, 2000), p. 194.

39 *Technê* and its actualization, i.e. “production” (*poiêsis*). The phrase “technical excellence” (*aretê technês*) appears at *EN* 1141a12.

40 Given that happiness needs a “complete life” (*bios teleios*; Aristotle, *EN*, 1098a19), and a premature death deprives life of happiness.

41 Aristotle, *EN*, 1198b15.

42 Broadie and Rowe, op. cit., p. 426.

43 Ibid., p. 426.

44 Aristotle, *EN*, 1177b15-21.

45 Such as in his account of moral habituation (Aristotle, Ibid., 1103b22).

46 Aristotle, Ibid., 1094a1-7

47 Aristotle, *Metaphysics* V.16.

48 Aristotle, *EN*, 1105a28-31.

49 Hirji, op. cit., p. 678. On this point, see also Margaret Hampson, “Imitating Virtue,” *Phronesis* 64.3 (2019): 292-320.

50 See Aristotle, *EN*, II.4.

51 See Hirji, op. cit.

52 See Jimenez, op.cit.

53 Aristotle, *EN*, 1161a16-18.

54 See, for instance, Aristotle, *EN*, 1145b11-14.

55 Aristotle, Ibid., 1098a7-8.

56 Aristotle, Ibid., 1098a13-15.

57 Aristotle, Ibid., 1097b26-27.

58 Christine Korsgaard’s distinction between descriptive and normative rationality is useful in this context: it is possible to be rational in the descriptive sense, insofar as we actualize the rational part of the soul, without thereby also being rational in the normative sense, i.e. by doing so virtuously. See Christine Korsgaard, *The Constitution of Agency: Essays on Practical Reason and Moral Psychology* (Oxford: Oxford University Press, 2008), p. 144.

59 “Decision is not possible without understanding and thought (*aneu nou kai dianoias*)” (Aristotle, *EN*, 1139a33-34). And it is not only acting well that requires thought, but also acting badly (*eupraxia kai to enantion*) (Ibid., 1139a34-35).

60 Aristotle, *EN*, 1098a10.

61 See Aristotle, *EN*, 1150a1-3.

62 Aristotle, *EE*, 1215a26-29.

63 Aristotle, Ibid., 1215a27.

64 Aristotle, *EN*, 1100b29-30.

65 Aristotle, Ibid., 1100b30-32.

